# Induction of ROS mediated genomic instability, apoptosis and G0/G1 cell cycle arrest by erbium oxide nanoparticles in human hepatic Hep-G2 cancer cells

**DOI:** 10.1038/s41598-022-20830-3

**Published:** 2022-09-29

**Authors:** Gehan Safwat, Esraa S. M. Soliman, Hanan R. H. Mohamed

**Affiliations:** 1 Zoology Department, Faculty of Science, Cairo University, Giza, Egypt; 2 Faculty of Biotechnology, October University for Modern Sciences and Arts, 6th of October, Egypt

**Keywords:** Cell biology, Ecology, Molecular biology, Medical research

## Abstract

The remarkable physical and chemical characteristics of noble metal nanoparticles, such as high surface-to-volume ratio, broad optical properties, ease of assembly, surfactant and functional chemistry, have increased scientific interest in using erbium oxide nanoparticles (Er_2_O_3_-NPs) and other noble metal nanostructures in cancer treatment. However, the therapeutic effect of Er_2_O_3_-NPs on hepatic cancer cells has not been studied. Therefore, the current study was conducted to estimate the therapeutic potential of Er_2_O_3_-NPs on human hepatocellular carcinoma (Hep-G2) cells. Exposure to Er_2_O_3_-NPs for 72 h inhibited growth and caused death of Hep-G2 cells in a concentration dependent manner. High DNA damage and extra-production of intracellular reactive oxygen species (ROS) were induced by Er_2_O_3_-NPs in Hep-G2 cells. As determined by flow cytometry, Er_2_O_3_-NPs arrested Hep-G2 cell cycle at the G0/G1 phase and markedly increased the number of Hep-G2 cells in the apoptotic and necrotic phases. Moreover, Er_2_O_3_-NPs caused simultaneous marked increases in expression levels of apoptotic (p53 and Bax) genes and decreased level of anti-apoptotic Bcl2 gene expression level in Hep-G2 cells. Thus it is concluded that Er_2_O_3_-NPs inhibit proliferation and trigger apoptosis of Hep-G2 cells through the extra ROS generation causing high DNA damage induction and alterations of apoptotic genes. Thus it is recommended that further in vitro and in vivo studies be carried out to study the possibility of using Er2O3-NPs in the treatment of cancer.

## Introduction

Erbium oxide is one of the most important rare earth metals and is widely used in various applications e.g. biomedicine because of its excellent optical, electrical and photoluminescence properties^1^. Nanomaterials doped with erbium oxide are of much interest because they have unique size-dependent optical and electrical properties. Erbium oxide doped nanoparticles are used in display monitors, synthesis of photoluminescence nanoparticles in 'green' chemistry^2^.

The distinctive physical and chemical properties of noble metal nanoparticles, including: high surface/volume ratio, wide optical properties, ease of assembly, surfactant and functional chemistry, have sparked scientific interest in the use of noble metal nanostructures in cancer treatments^3–5^. Erbium oxide nanoparticles (Er_2_O_3_-NPs) are used as gate dielectrics and coloring agents and also in biomedical applications e.g. bio-imaging^6^.

Hepatocellular carcinoma is the most common form of liver cancer, accounts for about 90% of cases and represents the third leading cause of cancer-related deaths worldwide^7,8^. Hepatocellular carcinoma may result from hepatic chronic inflammation, viral infection for example hepatitis B or C and exposure to toxins e.g. aflatoxins, alcohol, and pyrrolizidine alkaloids. Certain diseases e.g. alpha 1-antitrypsin deficiency and hemochromatosis, as well as, metabolic syndrome significantly increase the risk of hepatocellular carcinoma^9^. Several anticancer drugs e.g. bevacizumab, lenvatinib, cabozantinib, ramucirumab and regorafenib are now used for treatment of hepatocellular carcinoma. However, there is increasing growing research on finding alternative therapies for the used traditional chemotherapies because of its undesirable negative health effects such as fatigue, loss of appetite and increased liver enzymes^10^.

At present, cerium oxide nanoparticles doped with erbium ions have shown higher antioxidant and catalytic capabilities compared to cerium oxide nanoparticles^11^. Thus, the current study was conducted to estimate the effect of Er_2_O_3_-NPs on cell proliferation rate and genomic DNA integrity human hepatocellular carcinoma (Hep-G2) cells. The Sulforhodamine B (SRB) assay was performed to assess the effect of Er_2_O_3_-NPs on cell proliferation, while the alkaline comet assay was done to measure the level of DNA damage. Cell cycle analysis and apoptosis induction were also estimated using flow cytometry. The level of intracellular reactive oxygen species (ROS) was assessed using 2,7-dichlorofluorescein diacetate dye, and, further, the mRNA expression levels of apoptotic and anti-apoptotic genes were measured using real-time polymerase chain reaction.

## Materials and methods

### Chemicals

Erbium (III) oxide nanoparticles (Er_2_O_3_-NPs) were purchased from Sigma-Aldrich Chemical Company (Saint Louis, USA) with pink appearance and product number (203,238). Powders of Er_2_O_3_-NPs with 99.9 trace metals basis were suspended in deionized distilled water to prepare the required concentrations and ultra-sonicated prior use.

### Cell line

Human hepatocellular carcinoma (Hep-G2) cells were obtained from Nawah Scientific Inc., (Mokatam, Cairo Egypt). Cells were maintained in Dulbecco's Modified Eagle Medium (DMEM) media supplemented with streptomycin (100 mg/mL), penicillin (100 units/mL) and heat-inactivated fetal bovine serum (10) in humidified, 5% (v/v) CO2 atmosphere at 37 °C.

### Characterization of Er_2_O_3_-NPs

The purchased powders of Er_2_O_3_-NPs were characterized using a charge coupled device diffractometer (XPERT-PRO, PANalytical, Netherlands) to determine its X-ray diffraction (XRD) pattern. Zeta potential and particles' size distribution of Er_2_O_3_-NPs were also detected using Malvern Instrument Zeta sizer Nano Series (Malvern Instruments, Westborough, MA) equipped with a He–Ne laser (λ = 633 nm, max 5mW). Moreover, transmission electron microscopy (TEM) imaging was done to detect the shape and average particles' size of Er_2_O_3_-NPs suspension.

### Sulforhodamine B (SRB) cytotoxicity assay

Sulforhodamine B (SRB) assay was conducted to assess the influence of Er_2_O_3_-NPs on the proliferation of cancerous Hep-G2 cells^12^. Aliquots of 100 µl of Hep-G2 cells suspension containing 5 × 10^3^ cells were separately cultured in 96-well plates and incubated for 24 h in complete media. Hep-G2 Cells were then treated with five different concentrations of Er_2_O_3_-NPs (0.01, 0.1, 1, 10 and 100 µg/ml) incubated for 24 h or (0.1, 1, 10, 100 and 1000 µg/ml) incubated for 72 h. After 24 or 72 h of Er_2_O_3_-NPs exposure, cultured cells were fixed by replacing media with 10% trichloroacetic acid (TCA) and incubated for one hour at 4 °C. Cells were then washed five times with distilled water, SRB solution (0.4% w/v) was added and incubated cells in a dark place at room temperature for 10 min. All plates were washed three times with 1% acetic acid and allowed to air-dry overnight. Then, protein-bound SRB stain was dissolved by adding TRIS (10 mM) and the absorbance was measured at 540 nm using a BMG LABTECH-FLUO star Omega microplate reader (Ortenberg, Germany).

### Cells treatment

Cancerous Hep-G2 cells were cultured at the appropriate conditions and dived into control and treated cells. The control cells were treated with an equal volume of the vehicle (DMSO; final concentration, ≤ 0.1%), while the treated cells were treated with the IC50 of Er_2_O_3_-NPs. All cells were left for 72 h after nanoparticles treatment and were harvested by brief trypsinization and centrifugation. Each treatment was conducted in triplicate. Cells were washed twice with ice-cold PBS and used for different molecular assays.

### Estimation of genomic DNA integrity

The impact of Er_2_O_3_-NPs exposure on the integrity of genomic DNA in cancerous Hep-G2 cells was estimated using alkaline Comet assay^13,14^. Treated and control cells were mixed with low melting agarose and spread on clean slides pre-coated with normal melting agarose. After drying, slides were incubated in cold lysis buffer for 24 h in dark and then electrophoresed in alkaline electrophoresis buffer. Electrophoresed DNA was neutralized in Tris buffer and fixed in cold absolute ethanol. For analysis slides were stained with ethidium bromide, examined using epi-fluorescent microscope at magnification 200× and fifty comet nuclei were analyzed per sample using Comet Score software.

### Estimation of intracellular ROS generation

The effect of Er_2_O_3_-NPs exposure on intracellular ROS production in cancer Hep-G2 cells was studied using 2,7-dichlorofluorescein diacetate dye^15^. Cultured cells were washed with phosphate buffered saline (PBS) and then 2,7-dichlorofluorescein diacetate dye was added. Mixed cells and dye were left for 30 min in dark and spread on clean slides. The resultant fluorescent dichlorofluorescein complex from interaction of intracellular ROS with dichlorofluorescein diacetate dye was examined under epi-fluorescent at 20× magnification.

### Measuring the expression levels of apoptotic and anti-apoptotic genes

Quantitative real time Polymerase chain reaction (RT-PCR) was conducted to measure the mRNA expression levels of apoptotic (p53 and Bax) and anti-apoptotic (Bcl2) genes in control and treated Hep-G2 cells. Whole cellular RNA was extracted according to the instructions listed by the GeneJET RNA Purification Kit (Thermo scientific, USA) (Thermo scientific, USA) and using Nanodrop device purity and concentration of the extracted RNAs were determined. These RNAs were then reverse transcribed into complementary DNA (cDNA) using the instructions of the Revert Aid First Strand cDNA Synthesis Kit (Thermo scientific, USA). For amplification, RT-PCR was performed using the previously designed primers shown in Table 1^16,17^ by the 7500 Fast system (Applied Biosystem 7500, Clinilab, Egypt). A comparative *Ct* (DD*Ct*) method was conducted to measure the expression levels of amplified genes and GAPDH gene was used as a housekeeping gene. Results were expressed as mean ± S.D.

**Table 1:** Sequences of the used primers in qRT-PCR.

Gene	Strand	Primer's sequences
GAPDH	Forward	5′-GAAGGTGAAGGTCGGAGTCA-3′
	Reverse	5′-GAAGATGGTGATGGGATTTC-3′
BAX	Forward	5′-CCGCCGTGGACACAGAC-3′
	Reverse	5′-CAGAAAACATGTCAGCTGCCA-3′
BCL-2	Forward	5′-TCCGATCAGGAAGGCTAGAGT-3′
	Reverse	5′-TCGGTCTCCTAAAAGCAGGC-3′
P53	Forward	5′-CAGCCAAGTCTGTGACTTGCACGTAC-3′
	Reverse	5′-CTATGTCGAAAAGTGTTTCTGTCATC-3′

### Analysis of cell cycle distribution

Distribution of cell cycle was analyzed using flow cytometry. Control and treated cancer Hep-G2 cells with IC50 of Er_2_O_3_-NPs for 72 h were harvested, washed with PBS and re-suspended in 1 mL of PBS containing RNAase A (50 µg/mL) and propidium iodide (10 µg/mL) (PI). Cells were incubated for 20 min in dark at 37 C and analyzed for DNA contents using FL2 (λex/em 535/617 nm) signal detector (ACEA Novocyte flow cytometer, ACEA Biosciences Inc., San Diego, CA, USA). For each sample, 12,000 events are acquired and cell cycle distribution is calculated using ACEA NovoExpress software (ACEA Biosciences Inc., San Diego, CA, USA).

### Estimation of apoptosis induction

Apoptotic and necrotic cell populations were determined using Annexin V- Fluorescein isothiocyanate (FITC) apoptosis detection kit (Abcam Inc., Cambridge Science Park Cambridge, UK) coupled with two fluorescent channels flow cytometry. After treatment with Er_2_O_3_-NPs for 72 h and doxorubicin as a positive control, Hep-G2 cells were collected by trypsinization and washed twice with ice-cold PBS (pH 7.4). Harvested cells are incubated in dark with Annexin V-FITC/ propidium iodide (PI) solution for 30 min at room temperature, then injected via ACEA Novocyte flowcytometer (ACEA Biosciences Inc., San Diego, CA, USA) and analyzed for FITC and PI fluorescent signals using FL1 and FL2 signal detector, respectively (λex/em 488/530 nm for FITC and λex/em 535/617 nm for PI). For each sample, 12,000 events were acquired and positive FITC and/or PI cells are quantified by quadrant analysis and calculated using ACEA NovoExpress software (ACEA Biosciences Inc., San Diego, CA, USA).

### Statistical analysis

Results of the current study are expressed as mean ± Standard Deviation (S.D) and were analyzed using the Statistical Package for the Social Sciences (SPSS) (version 20) at the significance level *p* < 0.05. The *student t-test* was used to compare between the untreated and treated cancer Hep-G2 cells.

## Results

### Characterization of Er2O3-NPs

**Figure 1:**
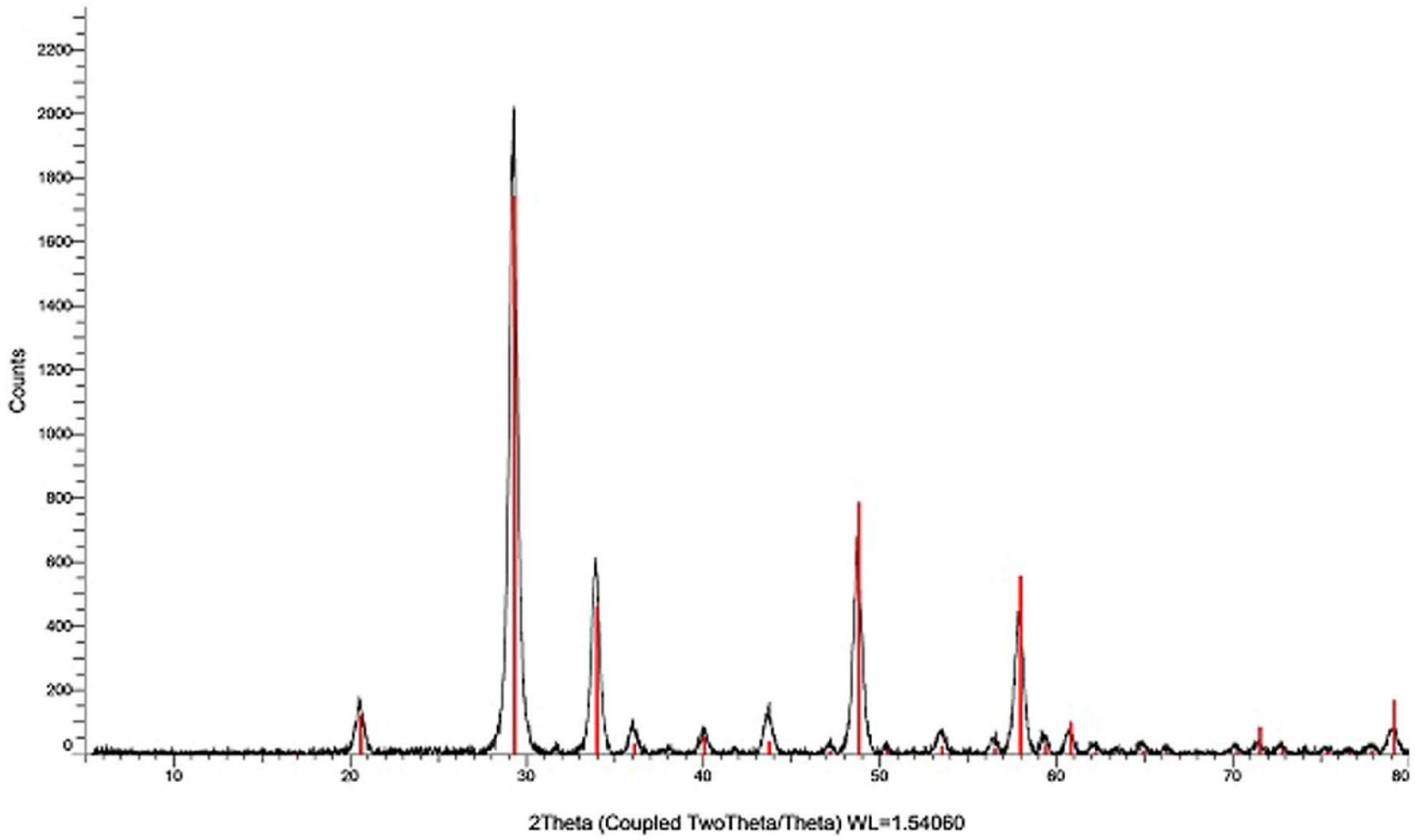
XRD pattern of Erbium Oxide nanoparticles.

**Figure 2:**
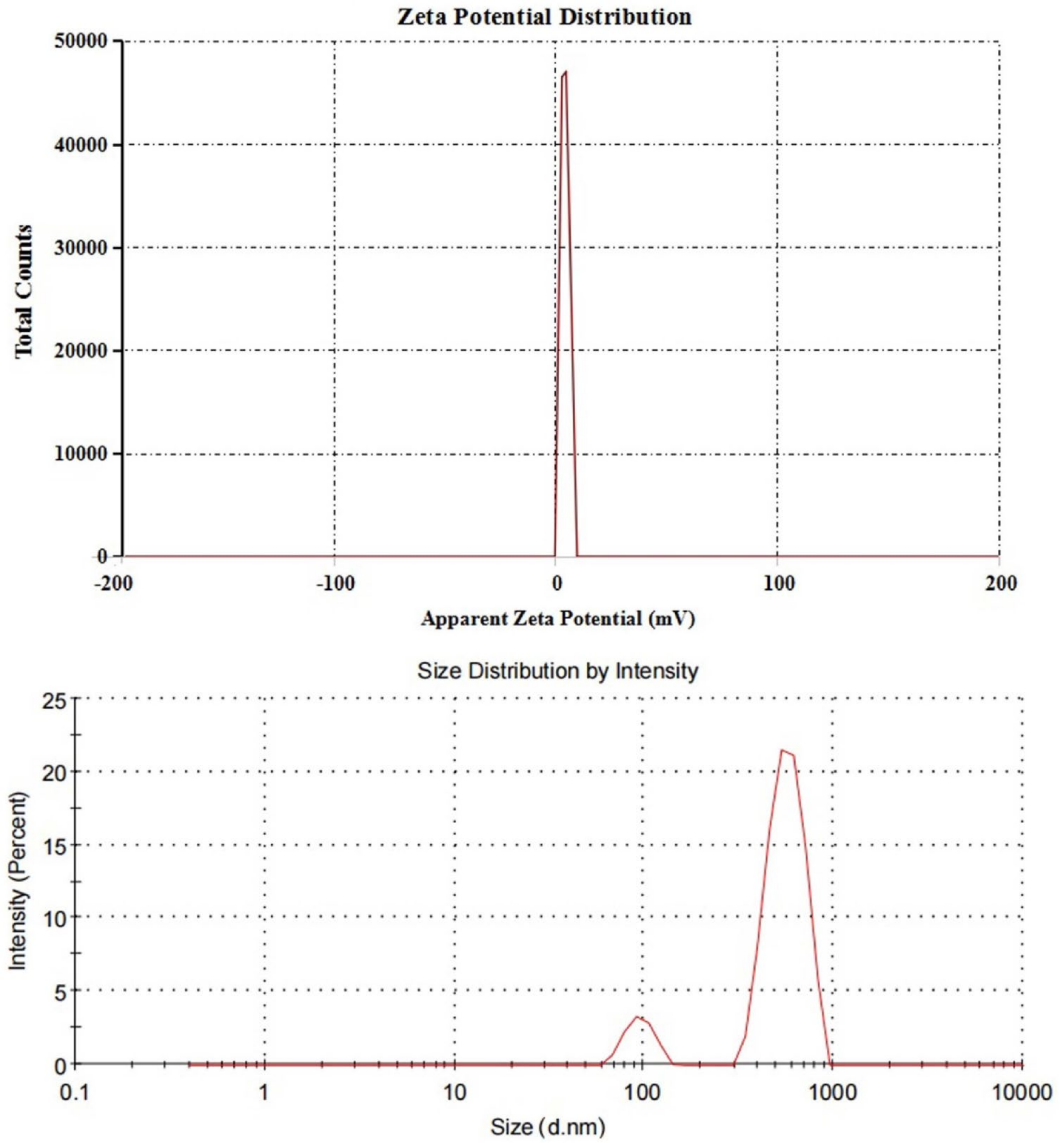
Zeta Potential and Size distribution of Erbium Oxide nanoparticles.

**Figure 3:**
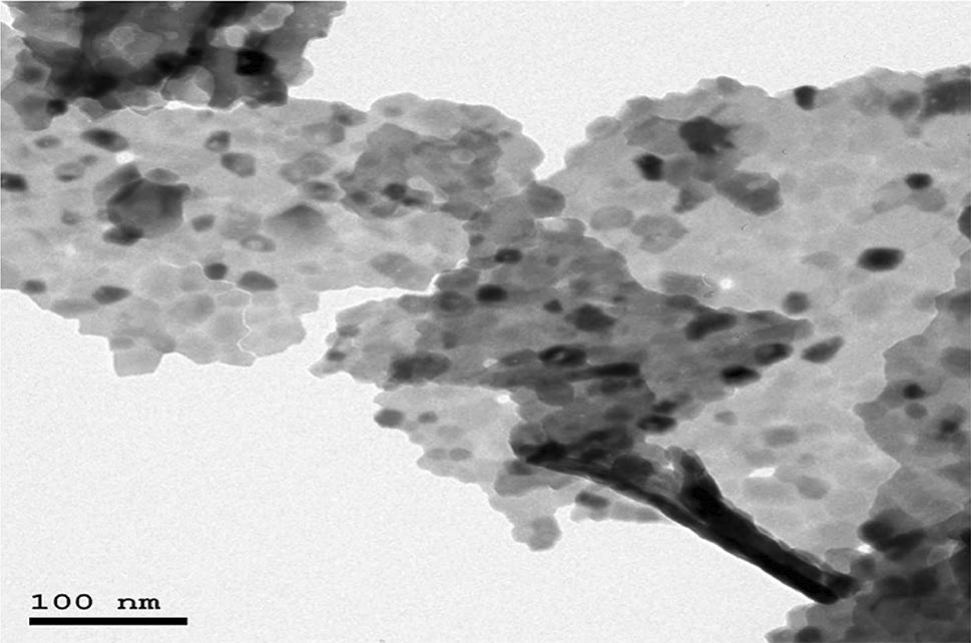
TEM image of Erbium Oxide nanoparticles.

The results of XRD analysis proved the purity of the purchased erbium oxide nano-powders through the appearance of distinct bands for Er_2_O_3_-NPs at diffraction angles of 29.33º, 33.99º, 48.84º and 58.00º (Fig. 1). However, the very low zeta potential of Er_2_O_3_-NPs of 3.21 mV was found to be insufficient for effective repletion among the suspended nanoparticles causing a high aggregation of suspended Er_2_O_3_-NPs in deionized distilled water and thus the particles' size of about 89.3% of the suspended Er_2_O_3_-NPs was found to be 570.7 nm and the remaining 10.7% of these nanoparticles were around 95.35 nm as displayed in Fig. 2. On the other hand, TEM imaging of the ultra-sonicated nanoparticles demonstrated the cubic shape and well dispersion of Er_2_O_3_-NPs with and average particles' size of 50 ± 3.55 nm. As a result, Er_2_O_3_-NPs were ultra-sonicated for 30 min prior treatment to ensure well dispersion of these nanoparticles (Fig. 3).

### Effect of Er2O3-NPs on cells viability

**Figure 4:**
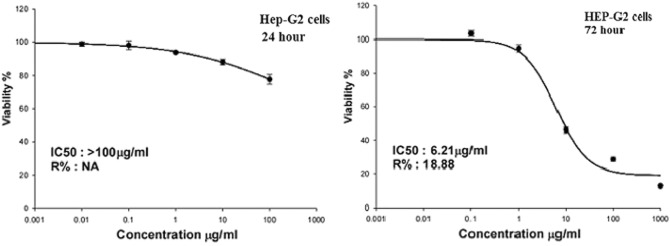
Viability of Hep-G2 cells after exposure to different concentrations of Erbium Oxide nanoparticles for 24 or 72 h.

Screening viability of Hep-G2 cells after exposure to five different concentrations of Er_2_O_3_-NPs (0.01, 0.1, 1, 10 and 100 µg/ml) for 24 h revealed that viability of Hep-G2 cells decreased slightly after treatment with Er_2_O_3_-NPs concentration greater than 0.1 µg/ml, and thus the half maximal inhibitory concentration (IC50) of Er_2_O_3_-NPs was found to be greater than 100 µg/ml in Hep-G2 cells (Fig. 4). By increasing the exposure time and concentrations of Er_2_O_3_-NPs to 72 h and to 1000 µg/ml, respectively, the viability of treated cancer Hep-G2 cells was highly decreased by increasing concentrations of Er_2_O_3_-NPs in a concentration dependent manner, and it was found that the IC50 of Er_2_O_3_-NPs is 6.21 µg/ ml as obvious in Fig. 4.

### Induction of genomic DNA damage

**Figure 5:**
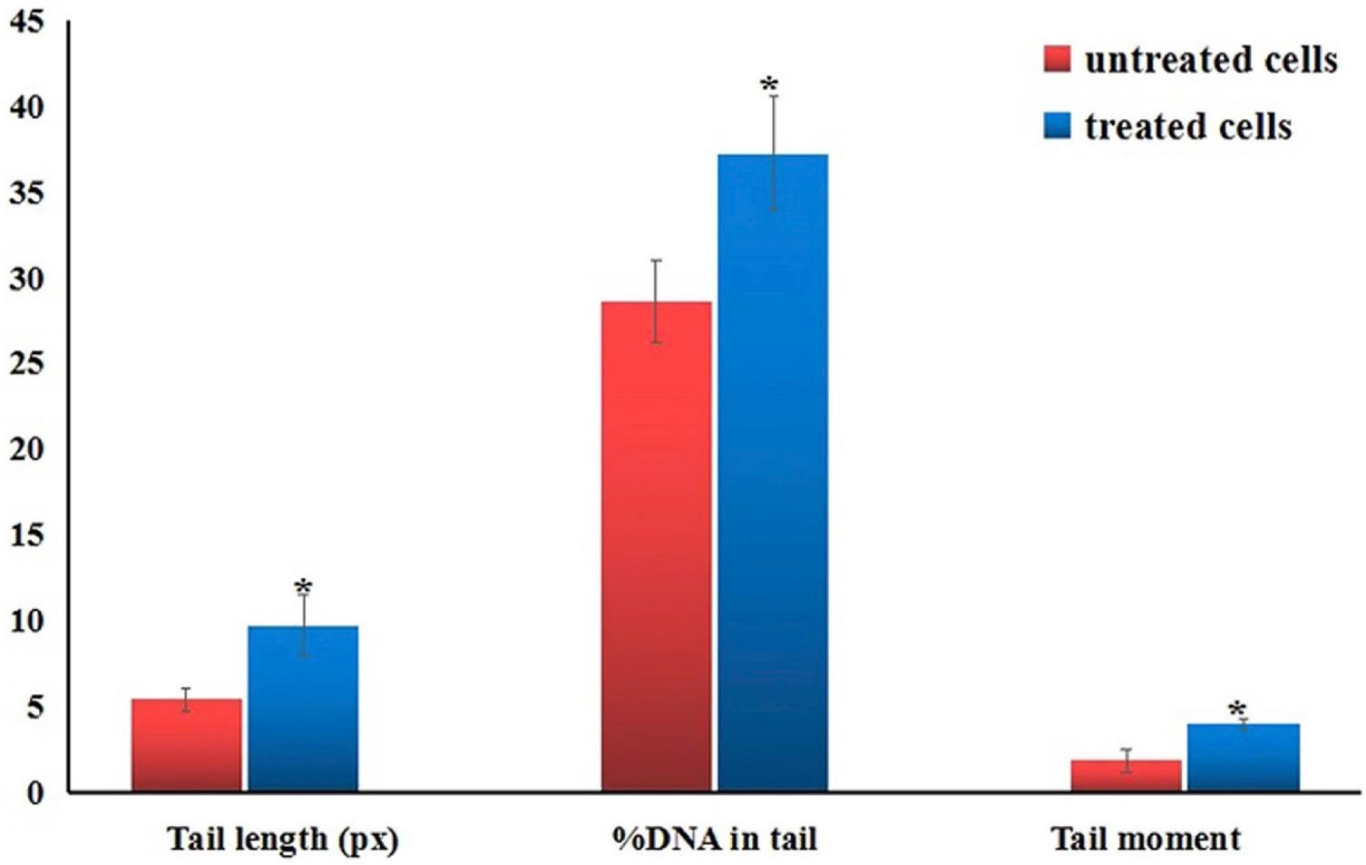
Tail length, %DNA in tail and tail moment in the control and treated Hep-G2 cells with IC50/72 h (6.21 µg/ml) of Erbium Oxide nanoparticles. Results are expressed as mean ± SD. *Indicates statistical significant difference from the compared control at *p* < 0.05 using *student t-test.*

**Figure 6:**
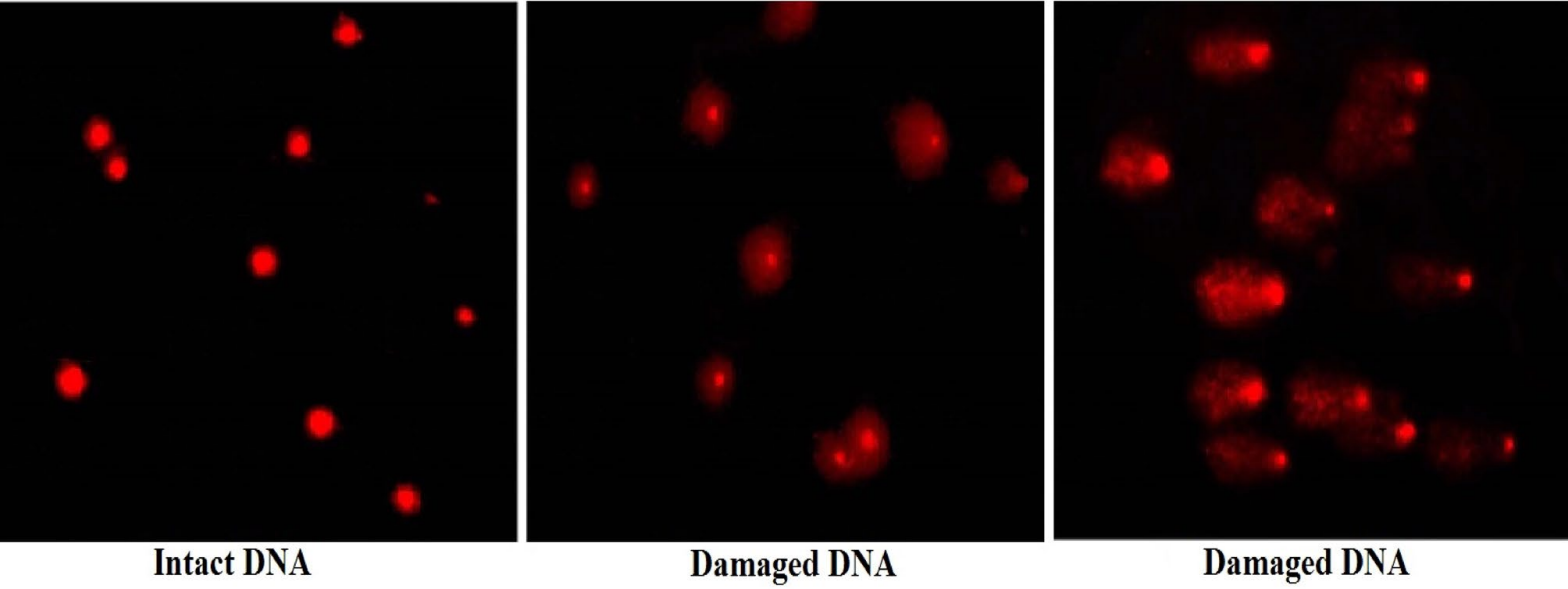
Representative examples for the scored Comet nuclei with intact DNA in untreated Hep-G2 cells and different degrees of DNA damage in Hep-G2 cells treated with 6.21 µg/ml of Erbium Oxide nanoparticles.

Comet assay results appeared that treatment of cancerous Hep-G2 cells with IC50 (6.21 µg/ml) of Er_2_O_3_-NPs resulted in statistically significant (*p* < 0.05) elevations in the DNA damage measured parameters: tail length, %DNA in tail and tail moment compared to untreated control Hep-G2 cells (Fig. 5). Examples for the scored Comet nuclei with different degrees of DNA damage are shown in Fig. 6.

### Generation of intracellular ROS

**Figure 7:**
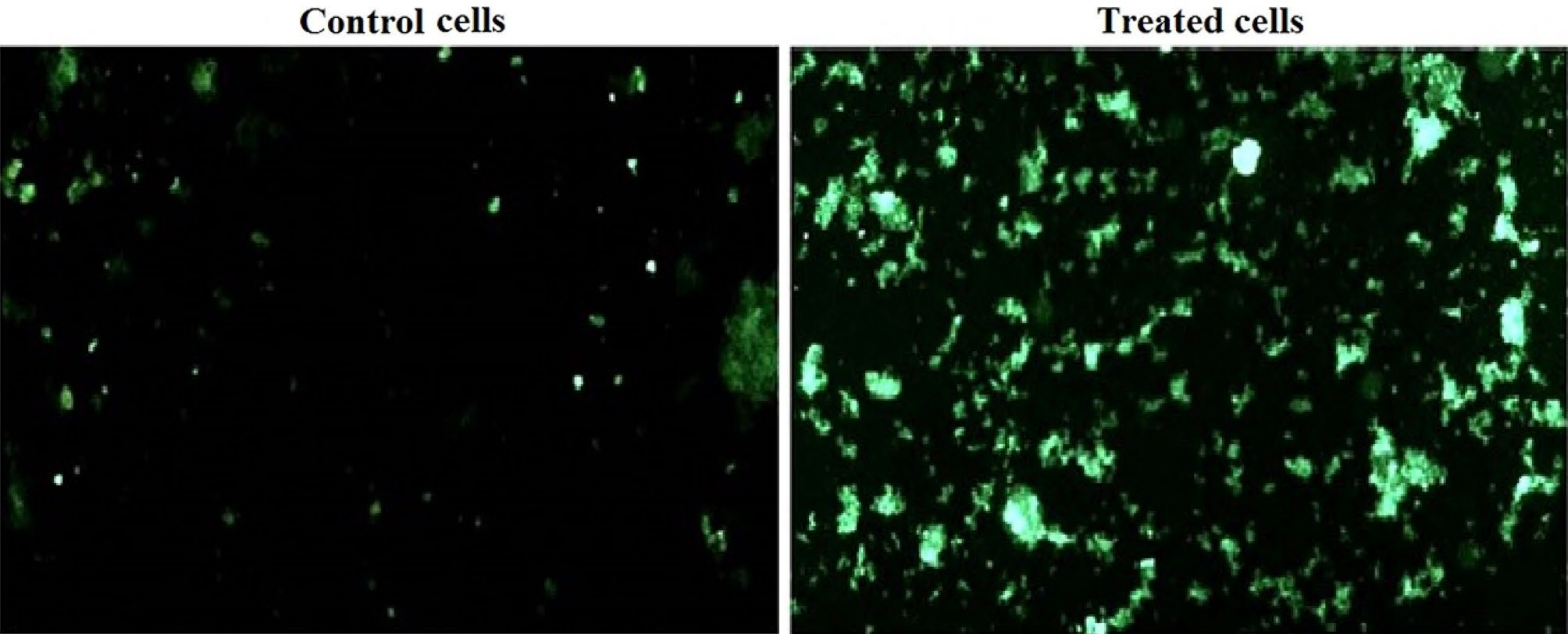
Intracellular ROS generation in the control and treated Hep-G2 cells with IC50/72 h (6.21 µg/ml) of Erbium Oxide nanoparticles.

As seen in Fig. 7 treatment of Hep-G2 cancer cells with IC50 (6.21 µg/ml) of Er_2_O_3_-NPs for 72 h caused high increases in intracellular ROS generation as manifested by the observed increases in the intensity of the florescent emitted light from the stained Hep-G2 cells with 2,7-dichlorofluorescein diacetate dye compared to the intensity of light emitted from the untreated Hep-G2 cells.

### Expression of apoptotic and anti-apoptotic genes

**Figure 8:**
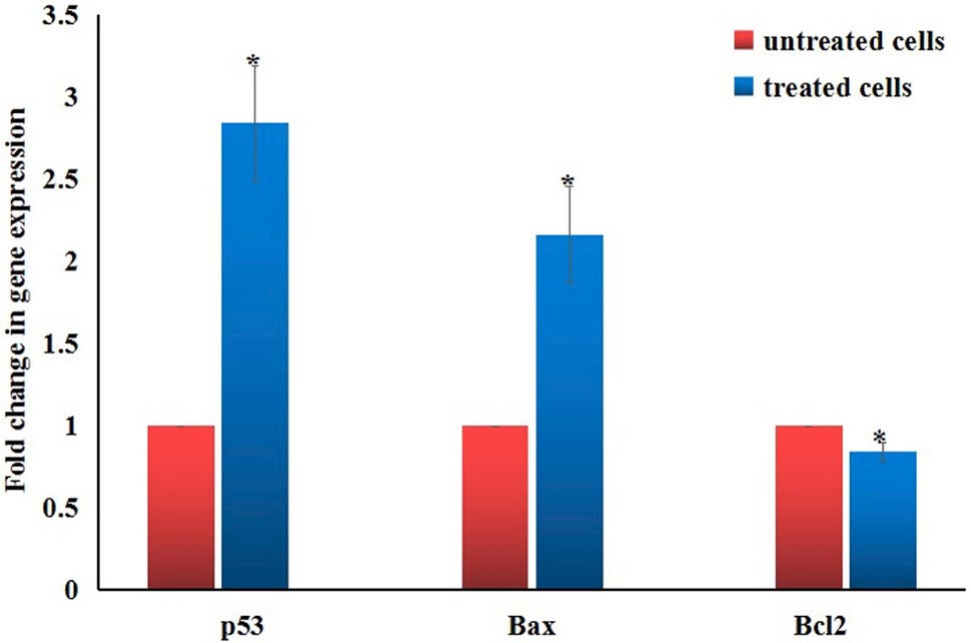
Expression levels of p53, Bax and Bcl2 genes in the control and treated Hep-G2 cells with IC_50_/72 h (6.21 µg/ml) of erbium oxide nanoparticles. Results are expressed as mean ± SD. *Indicates statistical significant difference from the compared control at *p* < 0.05 using *student t-test.*

Interpretation of RTPCR data revealed that exposure of Hep-G2 cancer cells to IC50 (6.21 µg/ml) of Er2O3-NPs for 72 h resulted in statistically significant (*p* < 0.05) elevations in the expression levels of the p53 and Bax (apoptotic) genes and decreases in the expression level of Bcl2 (anti-apoptotic) gene compared to their expression levels in the untreated Hep-G2 cells (Fig. 8).

### Cell cycle distribution

**Figure 9:**
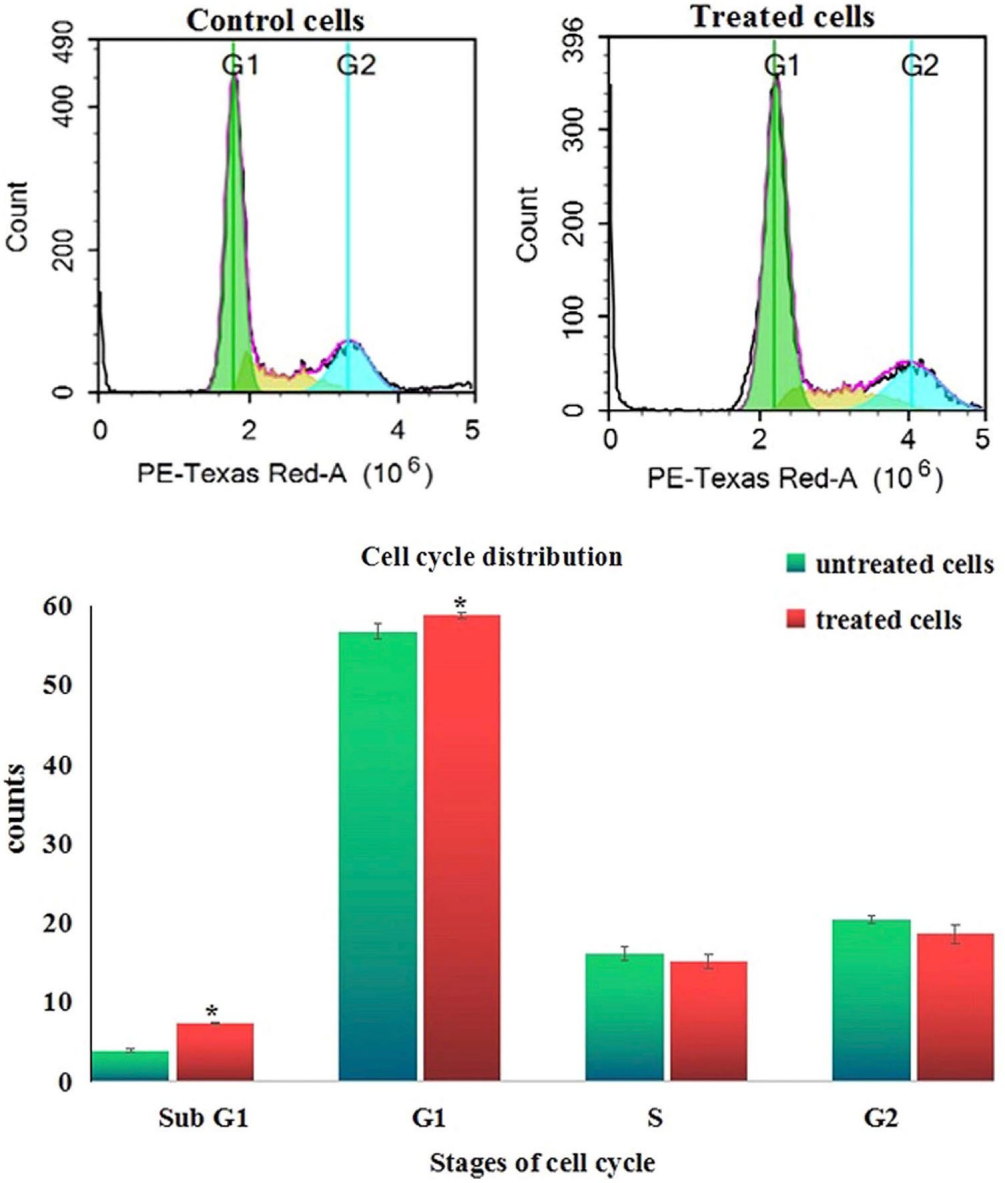
Cell cycle distribution of the control and treated Hep-G2 cells with IC_50_/72 h (6.21 µg/ml) of Erbium Oxide nanoparticles.

Analysis of cell cycle distribution appeared that exposure of Hep-G2 cancer cells to 6.21 µg/ml (IC50) of Er_2_O_3_-NPs for 72 h caused cell cycles arrest in the G0/G1 phase as manifested by the statistically significant (*p* < 0.05) observed increases in the number of Hep-G2 cells counts in the subG1 and G1 phases compared to untreated Hep-G2 populations in these phases of cell cycle (Fig. 9).

### Induction of apoptosis

**Figure 10:**
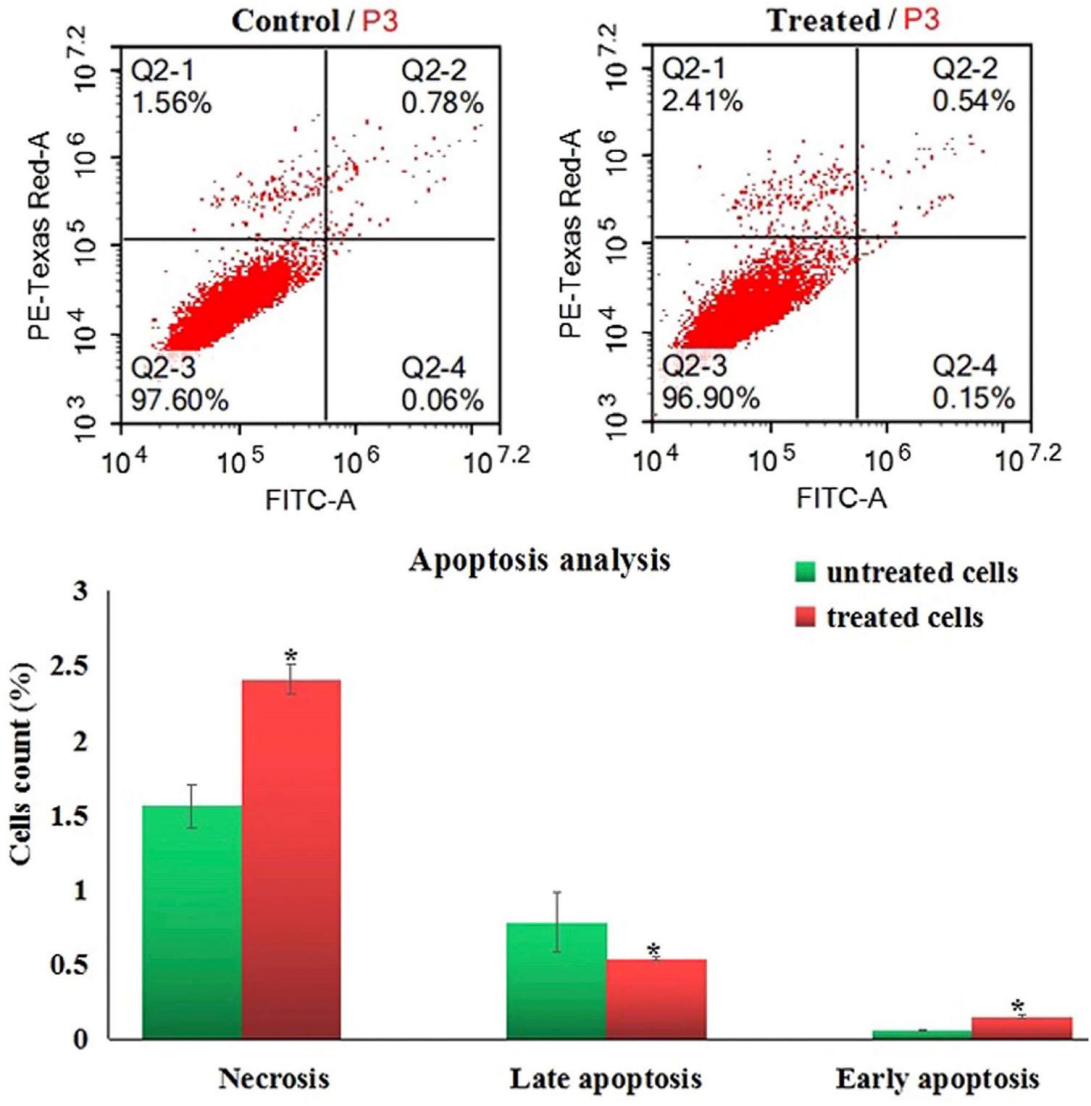
Induction of apoptosis in the control and treated Hep-G2 cells with IC_50_/72 h (6.21 µg/ml) of Erbium Oxide nanoparticles. Q2-1 denotes necrosis phase; Q2-2 denotes late apoptosis phase, Q2-3 denotes normal viable cells and Q2-4 denotes early apoptosis phase.

Screening apoptotic and necrotic cells using flow cytometry showed that treatment of Hep-G2 cancer cells with Er_2_O_3_-NPs at a concentration level of 6.21 µg/ml (IC50) for 72 h statistically increased the number of Hep-G2 cells in the early apoptotic and necrotic phases, meanwhile, the number of late apoptotic cells was highly decreased after exposure to Er2O3-NPs compared to untreated Hep-G2 cells at the same phases (Fig. 10).

## Discussion

The remarkable physical and chemical properties of rare-earth metals as well as the recently reported antioxidant and catalytic activities of erbium ions raise interest in studying the biomedical applications of erbium nanoparticles. Therefore, the current study was conducted to estimate the effect of Er_2_O_3_-NPs on proliferation rate and apoptosis induction in cancerous Hep-G2 cells.

First, the results of the SRB cytotoxicity assay proved the high toxicity of Er_2_O_3_-NPs as manifested by the remarkable proliferation inhibition and death of Hep-G2 cells noticed after exposure to Er_2_O_3_-NPs in a concentration-dependent manner. This cytotoxic effect may be attributed to the high increases in intracellular ROS production observed after exposure of cancerous Hep-G2 cells to Er_2_O_3_-NPs because increased ROS generation upsets the balance between oxidants and antioxidants that damage cellular lipids, proteins, carbohydrates, and even DNA^18,19^.

ROS are highly reactive and interact with genomic DNA causing severe DNA damage by inducing DNA breakages^20^. Our finding of high elevations in measured tail length, %DNA in tail and tail moment using Comet assay confirmed the inductions of DNA breaks by Er_2_O_3_-NPs in Hep-G2 cells. The alkaline Comet assay sensitively and accurately detects both single and double stranded DNA breaks in each cell separately, thus Er_2_O_3_-NPs induced both single- and double-stranded DNA breaks in Hep-G2 cells^13^.

Overproduction of ROS and induction of DNA breakage trigger apoptosis^21^. The induction of Hep-G2 apoptosis after Er_2_O_3_-NPs treatment was manifested in this study by the significant increases detected in the number of apoptotic and necrotic Hep-G2 cells using flow cytometry.

DNA breakage, especially double-stranded DNA breakage represent one of the most dangerous and deadly types of DNA damage because a single double-stranded DNA breakage is sufficient to disrupt the genetic integrity and kill the cell^22^. Consequently, detection of DNA damage triggers a cell response and induces p53 accumulation. The p53 gene, a tumor suppressor gene, has a pivotal role in the cell response to diverse stresses that cause DNA damage particularly ROS by mediating apoptosis through regulating the expression levels of the apoptotic Bax gene and the anti-apoptotic Bcl2 gene^23,24^.

In consistence with the aforementioned explanation, interpretation of the RTPCR data demonstrated that Er_2_O_3_-NPs induced apoptosis of Hep-G2 cells resulted from the noticed concurrent upregulation of the apoptotic (p53 and Bax) genes and downregulation of the anti-apoptotic (Bcl2) gene expression levels. Moreover, arresting of cells at the G0/G1 phase triggers apoptosis^25^, and thus the results of cell cycle analysis in this study confirmed the anti-proliferative effect of Er_2_O_3_-NPs by significant elevations in the number of Hep-G2 cells in G0/G1 phase detected by flow cytometry.

Regarding the safety of Er_2_O_3_-NPs on normal cells, contradictory findings shown by Mohamed and her colleagues^26^ of the high cytotoxicity and non-genotoxic effects of Er_2_O_3_-NPs towards normal human skin fibroblasts (HSF) cells are attributable to genomic stability and controlled DNA repair mechanisms, thus normal HSF cells maintain their genomic integrity.

## Conclusion

The results discussed above show that Er_2_O_3_-NPs inhibit cancerous Hep-G2 cells proliferation by causing cell cycle arrest in the G0/G1 phase and also induce apoptosis of Hep-G2 cancer cells through induction of DNA breakage, excessive intracellular ROS generation and upregulation of apoptotic genes. Therefore, further studies on different cell lines and in vivo animal models are recommended to study the possibility of using Er2O3-NPs in cancer treatment.

## Data Availability

The datasets used and/or analyzed during the current study are available from the corresponding author on reasonable request.
